# Effects of replacing hybrid giant napier with sugarcane bagasse and fermented sugarcane bagasse on growth performance, nutrient digestibility, rumen fermentation characteristics, and rumen microorganisms of Simmental crossbred cattle

**DOI:** 10.3389/fmicb.2023.1236955

**Published:** 2023-11-16

**Authors:** Yadong Jin, Yanru Huang, Haocen Luo, Langzhou Wang, Binlong Chen, Yi Zhang, Kaimei Deng, Ningbo Zhao, Anqiang Lai

**Affiliations:** ^1^College of Animal Science, Xichang University, Xichang, China; ^2^Sichuan Key Laboratory of Goats with Local Characteristics, Xichang, China; ^3^Ningnan County Rural Industry Technology Service Center, Liangshan, China

**Keywords:** Simmental crossbred cattle, fermented sugarcane bagasse, growth performance, nutrient digestibility, rumen bacteria

## Abstract

This study investigated the effects of replacing hybrid giant napiers with sugarcane bagasse and fermented sugarcane bagasse on the growth performance, apparent nutrient digestibility, rumen fermentation characteristics, and rumen microorganisms of Simmental crossbred cattle. Twenty-one Simmental crossbred cattle with similar initial body weight (363.42 ± 8.67 kg) were randomly divided into three groups: Group CON (20% hybrid giant napier +45% distillers grains +35% concentrate mixture), Group SB (20% sugarcane bagasse +45% distillers grains +35% concentrate mixture), and Group FSB (20% fermented sugarcane bagasse +45% distillers grains +35% concentrate mixture). The average daily weight gain in the SB group was lower than in the CON group, no significant difference was found between the CON and FSB groups. The feed conversion ratio of the CON and FSB groups was lower compared to the SB group. The apparent digestibility of neutral detergent fiber and acid detergent fiber in the SB group was lower than in the CON group, no significant difference was found between the CON and FSB groups. The levels of NH_3_-N, microbial protein, acetate, propionate, butyrate, isobutyrate, and total volatile fatty acids were higher in the CON and FSB groups than in the SB group, no significant difference was found between the CON and FSB groups. The relative abundances of *Christensenellaceae_R-7_group*, *Rikenellaceae_RC9_gut_group*, *Prevotellaceae_UCG-003*, *Saccharofermentans*, and *Eubacteriumcoprostanoligenes_group* were lower in the CON and FSB groups compared to the SB group. The relative abundance of *Succiniclasticum* was highest in the FSB group, followed by the CON group and then the SB group. Correlation analysis showed that the relative abundance of *Succiniclasticum* was positively correlated with propionate and NH_3_-N content, while the relative abundance of *Rikenellaceae_RC9_gut_group* was inversely correlated with NH_3_-N content. Gene function prediction indicated that fermented sugarcane bagasse promoted rumen microbial amino acid metabolism. In conclusion, replacing hybrid giant napiers with 20% sugarcane bagasse negatively affected the growth performance of Simmental crossbred cattle, while the addition of 20% fermented sugarcane bagasse had no adverse effects on growth performance and rumen fermentation characteristics, and did not alter the abundance of the rumen core flora in Simmental crossbred cattle.

## 1. Introduction

Sugarcane bagasse is the main byproduct of sugarcane production and an important renewable biological resource. China is the third-largest producer of sugarcane in the world, after Brazil and India, with an annual production of around 100 million tonnes [Food and Agriculture Organization of the United Nations (FAO), 2020]. Based on calculations that each tonne of sugarcane can yield 0.3 tonnes (wet basis) of sugarcane bagasse (Hofsetz and Silva, 2012), the sugar production in China generates approximately 30 million tonnes of sugarcane bagasse annually. Usually, sugarcane bagasse is burned inefficiently in a boiler as a fuel for sugar production. However, a high residual amount of sugarcane bagasse cannot be processed in a timely manner and is simply piled up, which can lead to serious air, water and soil pollution, as well as safety hazards such as fires (Meghana and Shastri, 2020). China’s animal livestock industry is currently undergoing rapid development, with a high increase in livestock numbers and a common tight supply of feed resources. If sugarcane bagasse is used to ruminant feeding, this concept can be an important means of achieving value-added and environmentally friendly management. However, animal digestion and nutrient utilization of sugarcane bagasse are limited by many factors, such as the low contents of crude protein (CP) [<3% dry material (DM)] and ether extract (EE, 0.3–1%), the high contents of cellulose (40–45% DM), hemicellulose (28–30%) and acid detergent lignin (ADL, 19–21%; Costa et al., 2015; de Almeida et al., 2018; So et al., 2020), as well as the cross-linking of ADL with cellulose and hemicellulose (Wanapat and Cherdthong, 2009; Raffrenato et al., 2017). *In vitro* simulations of the digestibility of sugarcane bagasse in the rumen revealed a DM degradation rate of only 23.90% after 48 h (Selim et al., 2022). The sugarcane harvesting season is usually from December to March–April of the following year, with a shortage of grass during winter and spring in southern China. Therefore, if sugarcane bagasse is improved to enhance its feeding value, it can effectively alleviate the shortage of forage for herbivores in the southern winter and spring, and reduce the pressure on the environment caused by unsustainable sugarcane bagasse treatment.

According to prior studies, the treatment of Sugarcane bagasse via physical, chemical, and biological methods can improve its forage value (Gunun et al., 2016; So et al., 2020). Microbial treatment is the most used method of the three options. Microbial fermentation not only reduces the content of cellulose, hemicellulose, and ADL in poor-quality roughage (So et al., 2020), but also increases the abundance of rumen cellulose-degrading bacteria (Hu et al., 2020), ultimately increasing the palatability, nutritional value, and storage time of the feed. Commonly used biofermentation strains include *Lactobacillus, Saccharomyces, Aspergillus niger, Bacillus*, and *Bradyrhizobium*, which increase the nutritional value of fermented feeds and improve the growth performance of animals (Jiang et al., 2020; Kholif et al., 2022; Mousa et al., 2022). Synergistic fermentation of bacteria and enzymes has a high fermentation efficiency, short fermentation cycle, and can improve the intestinal microecological environment of animals through the action of microorganisms and their metabolites, enhancing their resistance to diseases (So et al., 2021; Mousa et al., 2022). Previous studies have investigated the effects of different sugarcane-bagasse-based diets on growth performance, rumen fermentation and apparent digestibility of ruminants. However, the effects of sugarcane bagasse and fermented sugarcane bagasse on the rumen microbiota of ruminants have been seldom studied. We hypothesized that enzymatic treatment and microbial fermentation can enhance the nutritional quality of sugarcane bagasse, improve the rumen microbiota of ruminants. After the treatments, the sugarcane bagasse has high potential to replace roughage for beef cattle. Based on the hypothesis, in this study, we investigated the feasibility of fermented sugarcane bagasse as roughage for beef cattle using sugarcane bagasse with *Saccharomyces cerevisiae*, *Lactobacillus*, *Aspergillus niger*, *Aspergillus oryzae*, and cellulase for mycoenzymatic synergistic fermentation.

## 2. Materials and methods

### 2.1. Animal welfare statement

All animal experimental procedures were approved by the Welfare and Ethics Committee of the College of Animal Science at Xichang University (Approval No. xcc2022031).

### 2.2. Production method for fermented sugarcane bagasse

The sugarcane bagasse used in the experiment was purchased from the Ningnan County Sugar Extraction Factory, Liangshan Prefecture, Sichuan Province, and fermented sugarcane bagasse was obtained by fermentation with bacteria and enzymes. The strain was provided by Tianjin Yunli Star Co., Ltd., and the cellulase was provided by Hunan Hongyingxiang Co., Ltd. Sugarcane bagasse was crushed, cut with a forage cutter, and sieved through a 2 cm sieve. Based on dry matter, 0.3% strain combination (the strain combination was based on *Saccharomyces cerevisiae* (content ≥4 × 10^8^ CFU/g), *Aspergillus niger* (content ≥2 × 10^8^ CFU/g), *Aspergillus oryzae* (content ≥2 × 10^8^ CFU/g), and *Lactobacillus* (content ≥2 × 10^8^ CFU/g) prepared according to 2:1:1:1) and 0.1% cellulase (enzyme activity ≥20,000 IU/g) were added, while 0.5% urea was included in the dry matter calculation and added to the drinking water to adjust the moisture of the bagasse substrate to 38 ± 1%. Fermented sugarcane bagasse was prepared at a temperature of 20–30°C and sealed with a 200-L fermentation bucket. Air in the fermentation bucket was used for aerobic and then anaerobic fermentation. The fermented sugarcane bagasse was obtained at 96 h. The nutrient compositions of sugarcane bagasse and fermented sugarcane bagasse are listed in Table 1.

**Table 1:** Nutrient composition of sugarcane bagasse and fermented sugarcane bagasse (dry matter basis, %).

Items	Sugarcane bagasse	Fermented sugarcane bagasse
Dry matter	93.42	38.05
Crude protein	2.41	2.92
Ether extract	0.47	0.51
Crude fiber	43.46	32.99
Neutral detergent fiber	81.83	55.34
Acid detergent fiber	54.77	39.30
Lignin	13.50	10.71
Crude ash	3.76	4.04
Calcium	0.13	0.11
Phosphorus	0.02	0.06

### 2.3. Experimental animals and experimental design

A single-factor randomized design was used to test 21 Simmental crossbred cattle (weight, 363.42 ± 8.67 kg) in good body condition. The cattle were randomly divided into 3 treatment groups with 7 replicates per treatment and 1 beef cattle per replicate: CON group, 20% hybrid giant napier +45% distillers grains +35% concentrate mixture; SB group, 20% sugarcane bagasse +45% distillers grains +35% concentrate mixture; and FSB group, 20% fermented sugarcane bagasse +45% distillers grains +35% concentrate mixture. The experimental period was 87 days, including a 7d pre-feeding period and 80 days trial period. The cattle rations were designed according to the Institute of Animal Science of CAAS (2004) and Nutritional Requirements for Beef Cattle (2016). The ratio was a total mixed ration (TMR) with equal nitrogen levels. The nutritional composition of the feed is shown in Table 2.

**Table 2:** Composition and nutrient level of the base ration (dry matter basis).

Items	Experimental diets		
	CON	SB	FSB
Ingredient composition (%)			
Corn	23.40	23.05	23.05
Soybean meal	5.50	7.00	7.00
Corn gluten feed	2.30	0.50	0.50
Cottonseed meal	1.20	1.50	1.50
Slow-released urea	0.20	0.35	0.35
Distillers grains	45.00	45.00	45.00
Hybrid giant napier	20.00		
Sugarcane bagasse		20.00	
Fermented Sugarcane bagasse			20.00
limestone	0.90	0.90	0.90
CaHCO_3_	0.00	0.20	0.20
MgO	0.20	0.20	0.20
NaCl	0.32	0.32	0.32
NaHCO_3_	0.48	0.48	0.48
Premix^1^	0.50	0.50	0.50
Total	100	100	100
Nutrient content			
NE_mf_/(MJ/kg)^2^	9.54	9.07	9.15
Crude protein (%)	10.72	10.62	10.65
Ether extract (%)	2.74	2.53	2.55
Neutral detergent fiber (%)	45.04	48.20	43.09
Acid detergent fiber (%)	23.73	26.35	23.86
Acid detergent lignin (%)	1.67	4.38	3.81
Calcium (%)	0.48	0.47	0.48
Phosphorus (%)	0.32	0.30	0.31

^1^One kg of premix contained the following: VA 1600000 IU, VD3 60,000 IU, VE 6000 IU, Cu 2 g, Fe 12 g, Mn 8 g, Zn 8 g, Se 60 mg, I 120 mg, Co 40 mg. ^2^NEmf was a calculated value, NEmf = (NEm + NEg) × F, F was the correction coefficient for NEmf, reference to Feeding Standard of Beef Cattle (NY/T 815-2004), while the other nutrient levels were measured value.

### 2.4. Feeding management

The experiment was conducted from January 2022 to April 2022 at the Chen Qihong Cattle Breeding Cooperative, Huangshui Town, Xichang City. During the experiment, cattle were fed in a single pen twice daily (8:00 and 17:00). The feeding rate was set at 5% of the dry matter remaining in the trough. Water was provided *ad libitum* throughout the experiment. The barn was cleaned every morning after feeding and disinfected regularly.

### 2.5. Measurement indicators and methods

#### 2.5.1. Growth performance measurement

The initial and final weights of each cattle were measured at the beginning and end of the test period before morning feeding to calculate the ADG. ADG = (final weight - initial weight) / number of days in the experiment. During the trial period, daily feed intake and residual feed were recorded for each cattle to calculate the average dry matter intake (DMI). The F/G ratio (F/G) per cattle was calculated using ADG and DMI (F/G = DMI/ADG).

#### 2.5.2. Apparent digestibility of nutrients

The apparent digestibility of the diets was determined using an endogenous indicator method utilizing acid insoluble ash (AIA). Manure samples were collected 10 days before the end of feeding (manure samples were collected continuously for 5 days), and 100 g of fresh manure samples were collected from each cow through the rectum at 9:00 and 14:00 each day. As a result, 200 g of fresh manure sample was obtained. Thereafter, 20 mL of 10% sulfuric acid was added to the samples for nitrogen fixation. The samples were then stored at −20°C for measurement. The daily feed and remaining residues were weighed before the morning feeding during the 5 days of the digestibility trial. The dry matter (DM), CP, EE, Ash, Ca, and total phosphorus (TP) contents were determined according to the methods of AOAC (1995). The crude fiber (*CF*), neutral detergent fiber (NDF), and acid detergent fiber (ADF) contents were determined according to the method of Van Soest et al. (1991), and the total energy was determined using oxygen and nitrogen calorimetry (Shreck et al., 2017). The AIA was determined using 2 mol HCl (Van Keulen and Young, 1977). Apparent nutrient digestibilities were calculated based on the concentrations of AIA and nutrients in diets fed, orts, and feces (Holt et al., 2010). The apparent digestibility of feed nutrients was calculated as follows:


$$\begin{array}{l} \mathrm{Apparent}\;\mathrm{digestibility}\;\mathrm{of}\;\mathrm{feed}\;\mathrm{nutrients}\;\left(\%\right)\\ \;=100-100\times \left(a\times B/A\times b\right) \end{array}$$

where A is the nutrient content of the ratio (%), a is the nutrient content of the manure sample (%), B is the AIA content of the ratio (%), and b is the AIA content of the manure sample (%).

#### 2.5.3. Measurement of the rumen fermentation parameters

At the end of the experiment, four cattle were randomly selected, and approximately 50 mL rumen fluid were extracted from the rumen using a rumen collector through the mouth 4 h after morning feeding. The pH was immediately measured on site with a BU-7 acidity meter. Then, the ruminal fluid samples were filtered through four layers of cheesecloth and divided into four portions. The first 10 mL of ruminal fluid sample was mixed with 2 mL of 20 g/L (w/v) H_2_SO_4_ and froze at −20°C for NH_3_-N analysis using the method described by Chaney and Marbach (1962). The second 10 mL of ruminal fluid sample was frozen at −20°C for future analysis of Microbial protein (MCP) according to Verdouw et al. (1978). The third 20 mL sample of ruminal fluid was kept in a centrifuge tube and frozen at−80°C for DNA extraction. The fourth 10 mL sample of ruminal fluid was used for volatile fatty acid (VFA) analysis. VFA was determined using gas chromatography (GC-2010 Plus, Shimadzu, Japan). The sample was pre-treated (on ice throughout) as follows: the supernatant was aspirated in a 2 mL centrifuge tube and then centrifuged at 10000 r/min for 10 min at 4°C. The supernatant was collected (at least 2 mL for each sample) and 1.280 mL of the sample filtrate was aspirated. Following the addition of 0.240 mL of internal standard solution, 0.480 mL of metaphosphate was added to a 2 mL centrifuge tube and allowed to stand on ice for 30 min. The sample mixture was centrifuged at 10000 r/min for 10 min at 4°C, and the supernatant was filtered through a 0.45 μm membrane in a sample vial and injected into a full autosampler for detection. Acetate, propionate, butyrate, valerate, isobutyrate, and isovalerate (98.00% chromatographic purity) were purchased from Guizhou Dida Technology Co. Ltd. The chromatographic conditions were as follows: column, SH-Rtx-WAX (30.00 m × 0.25 mm × 0.25 μm); carrier gas, high-purity nitrogen; and splitting ratio, 50:1. The column temperature was set to 100°C for 2 min and increased to 150°C at 5°C/min for 2 min. The flame hydrogen ion detector (FID) temperature was 240°C. The temperature of the gas chamber was 220°C, and the injection volume was 1 μL. Crotonic acid was used as the internal standard.

#### 2.5.4. Determination of rumen microflora

The samples were sent to Guangzhou Kidio Biotechnology Co., Ltd. to determine the rumen microbiota. DNA was extracted using the microbial DNA extraction kit, and the conserved regions of 16S rDNA V3-V4 and V4 were amplified with specific primers with barcode. The sequences of the 16S rDNA V3-V4 specific primers are 341F (5´-CCTAGGGNGGCWCAG-3′) and 806R (5´-GGACTACHVG GGTATCTAAT-3′), and the 16S rDNA V4 specific primer sequences are 515F (5´-GTGYCAGCMGCCCGGTAA-3′) and 806R (5´-GGA CTACNVGGGTWTCTAAT-3′). The PCR amplification products were recovered via gel cutting and quantified using a QuantiFluor™ fluorometer. The purified amplification products were mixed in equal amounts, the sequencing connectors were attached, and sequencing libraries were constructed and sequenced on an Illumina PE250 platform.

The fragments from high-throughput sequencing were spliced using Fastp Flash software and quality-controlled using QIME. Chimeras were removed using the UCHIME algorithm and Gold database to obtain limited fragments (effective reads). Based on the USEARCH^1^ software, the UPARSE algorithm was used to perform operational taxonomic unit (OTU) clustering at a 97% agreement level, and the most frequently occurring sequences in OTUs were selected as representative OTUs. The obtained downstream data were homogenized for each sample, and alpha diversity, beta diversity classification, and BugBase phenotype classification were predicted.

### 2.6. Data processing and statistical methods

Growth performance, rumen fermentation parameters, apparent nutrient digestibility, OTU number, alpha diversity, and relative abundance of rumen microorganisms at the phylum level and genus level with mean abundance greater than or equal to 1% were analyzed via the GLM procedure of SAS 9.2. The results are presented as mean values and standard errors of the mean deviation. The differences between groups were determined by Duncan’s Multiple Range Test Treatment differences were considered statistically significant at *p* < 0.05. Correlations between rumen fermentation parameters and rumen bacterial genera with a mean relative abundance greater than 1% were analyzed using Spearman’s correlation coefficient. Linear discriminant analysis effect sizes were analyzed using the LEFse software and LDA score thresholds greater than 4. KEGG function predictions were performed using Tax4Fun for the three treatments with SILVA annotation of the 16S sequences, and abundance tables for each treatment were provided and analyzed by Guangzhou Kidio Biotechnology Co.

## 3. Experimental results

### 3.1. Effect on growth performance

As shown in Table 3, no significant differences were found between the initial and final weights of the Simmental crossbred cattle in each group (*p* > 0.05). The DMI of ruminants in the CON group was significantly higher than that of ruminants in the SB group (9.29 vs. 8.78 kg/d) and FSB group (9.29 vs. 9.06 kg/d), and that of the FSB group was significantly higher than that of the SB group (9.06 vs. 8.78 kg/d; *p* < 0.05). The ADG in the CON group was significantly higher than that in the SB group (0.98 vs. 0.84 kg/d; *p* < 0.05). The FCR was significantly higher in the SB group than in the CON (10.43 vs. 9.49) and FSB groups (10.43 vs. 9.65; *p* < 0.05).

**Table 3:** Effect of bagasse and fermented sugarcane bagasse feeding on the growth performance of beef cattle.

Items^1^	CON	SB	FSB	SEM	*p*-value^2^
Initial weight (kg)	362.59	363.59	364.08	1.89	0.952
Final weight (kg)	440.90	430.77	439.21	2.10	0.105
DMI (kg/d)	9.29^a^	8.78^b^	9.06^c^	0.09	<0.001
ADG (kg/d)	0.98^a^	0.84^b^	0.94^ab^	0.01	0.030
FCR (kg DMI/kg ADG)	9.49^a^	10.43^b^	9.65^a^	0.10	<0.001

^1^DMI, dry matter intake; ADG, average daily gain; FCR, feed conversion ratio. ^2^Differences between means were considered significant when the *p*-value of the ANOVA *F*-test was less than 0.05. ^a–c^Mean values in the same row with different superscripts are significantly different (*p* < 0.05).

### 3.2. Effect on nutrient digestibility

The apparent digestibilities of dry matter, organic matter, and crude protein were significantly higher in the CON and FSB groups than in the SB group (*p* < 0.05). The apparent digestibility of DNF was significantly higher in the CON group than in the SB group (50.16 vs. 43.63%; *p* < 0.05), no significant difference was found between CON and FSB groups (50.16 vs. 46.97%), as well as the FSB and SB groups (46.97 vs. 43.63%; *p* > 0.05). The apparent digestibility of ADF was significantly lower in the SB group than in the CON (38.33 vs. 42.16%) and FSB groups (38.33 vs. 41.49%; *p* < 0.05), no significant difference was found between CON and FSB groups (42.16 vs. 41.49%; *p* > 0.05; Table 4).

**Table 4:** Effect of bagasse and fermented sugarcane bagasse feeding on apparent digestibility of nutrients in beef cattle (%).

Items^1^	CON	SB	FSB	SEM	*p*-value^2^
DM	64.42^a^	55.51^b^	60.61^a^	1.14	0.002
OM	64.94^a^	56.36^b^	61.04^a^	1.13	0.003
CP	53.11^a^	39.88^b^	51.43^a^	1.53	<0.001
EE	70.50	69.09	70.95	0.94	0.723
NDF	50.16^a^	43.63^b^	46.97^ab^	0.87	<0.004
ADF	42.16^a^	38.33^b^	41.49^a^	0.66	<0.031

^1^DM, dry matter; OM,organic matter; CP,crude protein; EE, ether extract; NDF, neutral detergent fiber; ADF, acid detergent fiber. ^2^Differences between means were considered significant when the *p*-value of the ANOVA *F*-test was less than 0.05. ^a–c^Mean values in the same row with different superscripts are significantly different (*p* < 0.05).

### 3.3. Effect on rumen fermentation characteristics

As shown in Table 5, the rumen liquor of the SB group contained NH_3_-N, MCP, acetate, propionate, butyrate, isobutyrate, and TVFA in the rumen fluid of SB, with levels significantly lower than those in the CON and FSB groups (*p* < 0.05). No significant difference was found between the CON and FSB groups (*p* > 0.05). The pH value of rumen fluid in the SB and FSB groups tended to be higher than that in the CON group (*p* = 0.054), with that in the SB group being slightly higher than that in the FSB group.

**Table 5:** Effect of bagasse and fermented sugarcane bagasse feeding on the rumen fermentation characteristics of beef cattle (%).

Items	CON	SB	FSB	SEM	*p*-value^2^
pH	6.35	6.48	6.43	0.02	0.054
NH_3_-N (mg/dl)	12.90^a^	11.97^b^	13.02^a^	0.19	0.027
MCP (mg/mL)	6.35^a^	5.35^b^	6.03^a^	0.16	0.012
Acetate (mmol/L)	56.86^a^	49.89^b^	55.49^a^	1.19	0.020
Propionate (mmol/L)	13.42^a^	11.53^b^	12.74^a^	0.29	0.007
Acetate/Propionate	4.24	4.32	4.23	0.06	0.878
Isobutyrate (mmol/L)	0.39^a^	0.33^b^	0.39^a^	0.01	0.045
Butyrate (mmol/L)	6.71^a^	5.18^b^	6.63^a^	0.27	0.014
Isovalerate (mmol/L)	0.46	0.47	0.50	0.02	0.810
Valerate (mmol/L)	0.48	0.43	0.43	0.02	0.576
TVFA (mmol/L)	78.33^a^	67.84^b^	76.18^a^	1.69	0.009

^1^MCP, microbial protein; TVFA, total volatile fatty acids. ^2^Differences between means were considered significant when the *p*-value of the ANOVA *F*-test was less than 0.05. ^a-b^Mean values in the same row with different superscripts are significantly different (*p* < 0.05).

### 3.4. Effect on the diversity of rumen bacteria alpha

A total of 1,059,006 valid sequences were obtained from 12 rumen samples after quality control and chimera removal, with an average of 88,251 ± 5,245 sequences per sample. Based on the 97% sequence similarity, a total of 28,641 OTUs were identified (2,387 ± 211 per sample). The percentage of good coverage averaged 98.97% across all samples, indicating adequate sequence coverage. As shown in Table 6, the number of OTU in the FSB group was significantly lower than those in the CON and SB groups (*p* < 0.05), and the number of OTU in the SB group was slightly higher than that in the CON group, with no significant difference between the two groups (*p* > 0.05). The Chao1 index of the SB group was slightly higher than that of the CON group (*p* > 0.05). The ACE index of the FSB group was significantly lower than that of the SB group (*p* < 0.05) and the ACE index of the SB group was slightly higher than that of the CON group (*p* > 0.05).

**Table 6:** Effect of bagasse and fermented sugarcane bagasse feeding on the alpha diversity of rumen bacteria.

Items	CON	SB	FSB	SEM	*p*-value^1^
OUTs	2457.75^a^	2562.50^a^	2140.00^b^	63.67	0.003
Shannon index	8.43	8.59	8.04	0.10	0.069
Simpson index	0.99	0.98	0.98	0.00	0.402
Chao1 index	2528.12^a^	2633.23^a^	2206.40^b^	63.77	0.002
ACE index	2622.79^ab^	2734.71^a^	2297.29^b^	65.01	0.002
Goods_coverge/%	98.97	98.93	99.03	0.00	0.247

^1^Differences between means were considered significant when the *p*-value of the ANOVA *F*-test was less than 0.05. ^a-b^Mean values in the same row with different superscripts are significantly different (*p* < 0.05).

### 3.5. Effect on the abundance of rumen bacteria gates

At the phylum level, 12 phyla with an average relative abundance greater than 0.1% were detected (Table 7). The relative abundance of Kiritimatiellaeota in the FSB group was significantly lower than that in the SB group (*p* < 0.05), whereas the abundance of Kiritimatiellaeota in the CON group was not significantly different from that in the SB and FSB groups (*p* > 0.05). The relative abundance of Kiritimatiellaeota in the SB group was the highest among the three groups and was significantly higher than that in the CON group (*p* < 0.05).

**Table 7:** Effect of bagasse and fermented sugarcane bagasse feeding on rumen bacterial gate abundance.

Items	CON	SB	FSB	SEM	*p*-value^1^
Firmicutes	50.97	48.75	48.15	1.15	0.622
Bacteroidetes	31.76	37.84	34.91	1.60	0.328
Euryarchaeota	6.26	3.36	7.97	1.10	0.238
Patescibacteria	2.55	2.16	1.68	0.21	0.253
Actinobacteria	0.81	0.45	0.61	0.08	0.245
Proteobacteria	1.70	1.29	0.89	0.16	0.119
Spirochaetes	0.74	0.45	1.32	0.19	0.180
Planctomycetes	0.61^ab^	0.98^a^	0.50^b^	0.09	0.042
Tenericutes	0.59	0.31	0.48	0.06	0.143
Kiritimatiellaeota	0.17^a^	0.46^b^	0.26^ab^	0.05	0.040
Chloroflexi	0.21	0.26	0.15	0.03	0.435
Fibrobacteres	0.13	0.06	0.14	0.02	0.094
Synergistetes	0.03	0.08	0.03	0.01	0.113

^1^Differences between means were considered significant when the *p*-value of the ANOVA *F*-test was less than 0.05. ^a-b^Mean values in the same row with different superscripts are significantly different (*p* < 0.05).

### 3.6. Effect on the abundance of rumen bacterial genera

At the genus level, 16 phyla with an average relative abundance of >1% were detected (Table 8). As shown in Table 8, *Christensenellaceae_R-7_group*, *Rikenellaceae_RC9_gut_group, Prevotellaceae_UCG-003, Saccharofermentans,* and *Eubacterium_coprostanoligenes_group* were significantly more abundant in the SB group than in the CON and FSB groups (*p* < 0.05). Among the three groups, *Succiniclasticum* abundance was highest in the FSB group and lowest in the SB group, with significant differences among all three groups (*p* < 0.001).

**Table 8:** Effect of bagasse and fermented sugarcane bagasse feeding on the abundance of rumen bacteria.

Items	CON	SB	FSB	SEM	*p*-value^1^
Christensenellaceae_R-7_group	8.68^a^	13.43^b^	9.42^a^	0.89	0.045
Prevotella_1	7.67	8.84	10.35	0.82	0.446
Rikenellaceae_RC9_gut_group	7.61^a^	10.90^b^	6.40^a^	0.69	0.005
Succiniclasticum	6.85^a^	3.48^b^	8.60^c^	0.69	<0.001
Methanobrevibacter	6.18	3.31	7.86	1.09	0.240
Ruminococcus_2	3.05	3.57	2.81	0.25	0.501
Ruminococcaceae_NK4A214_group	2.59	2.29	2.03	0.15	0.339
Candidatus_Saccharimonas	2.41	1.81	1.39	0.22	0.156
Lachnospiraceae_NK3A20_group	1.67	2.25	1.54	0.21	0.358
Prevotellaceae_UCG-003	1.38^a^	2.44^b^	1.50^a^	0.18	0.015
Lactobacillus	4.98	0.09	0.03	1.01	0.052
Saccharofermentans	1.29^a^	2.12^b^	1.45^a^	0.15	0.028
Ruminococcaceae_UCG-014	1.49	1.45	1.31	0.13	0.865
Prevotellaceae_UCG-001	0.98	1.37	1.30	0.11	0.309
Butyrivibrio_2	1.14	0.79	1.43	0.13	0.119
Eubacterium_coprostanoligenes_group	0.87^a^	1.32^b^	0.96^a^	0.08	0.012

^1^Differences between means were considered significant when the *p*-value of the ANOVA *F*-test was less than 0.05. ^a-b^Mean values in the same row with different superscripts are significantly different (*p* < 0.05).

### 3.7. Indicator species analysis

**Figure 1:**
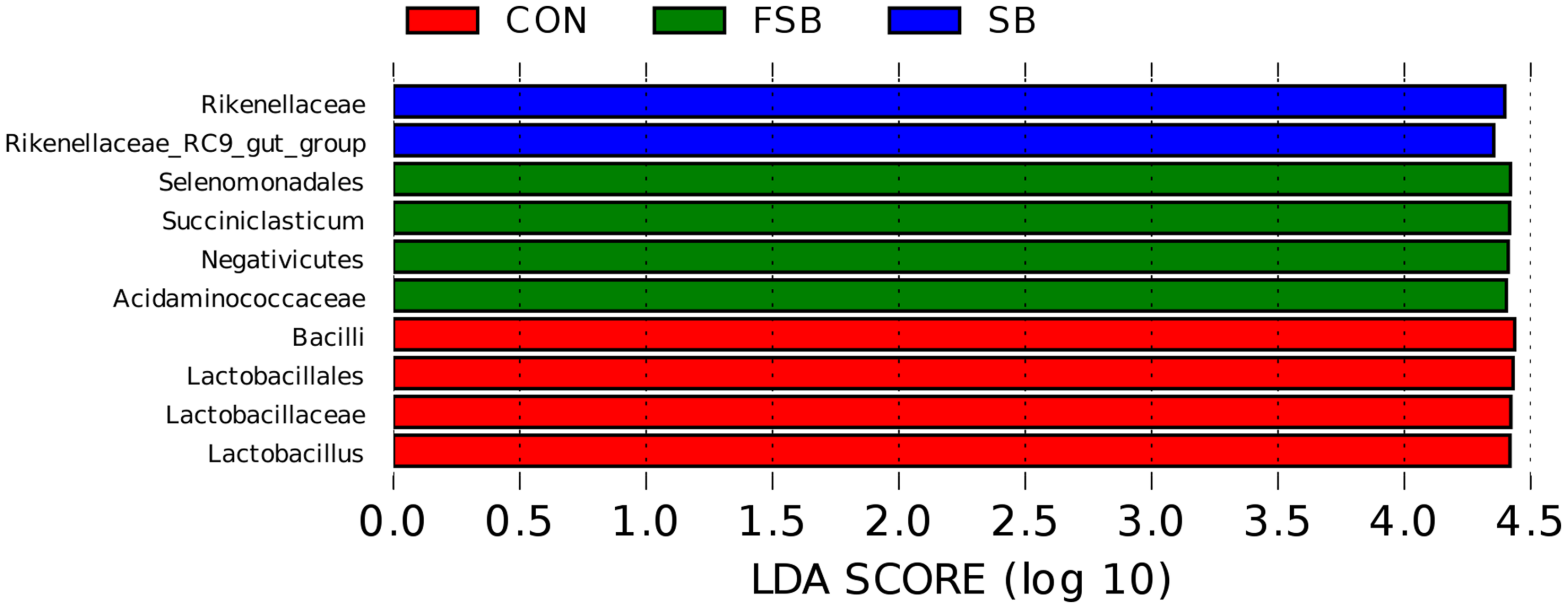
Analysis of LDA effect size (LEfSe) of rumen bacteria in the CON, SB, and FSB groups. The LDA score represents the difference in relative abundance between the three treatments with an exponential fold change of 10 and LDA score > 4.0.

To better understand the differential bacteria among the CON, SB, and LC groups, LEFse software was used to analyze the differential species between the groups (Figure 1). Figure 1 shows a representative taxonomic hierarchy of the main microbiome structures, indicating that the taxonomic hierarchy of species differed significantly among the three groups. A total of 10 taxonomic classes with significant differences were detected in this study, including four in the CON group, two in the SB group, and four in the FSB group. The abundance of *Rikenellaceae* and *Rikenellaceae_RC9_gut_group* was higher in the FSB group than in the CON and FSB groups.

### 3.8. Correlation between rumen fermentation parameters and abundance of bacterial genera

**Figure 2:**
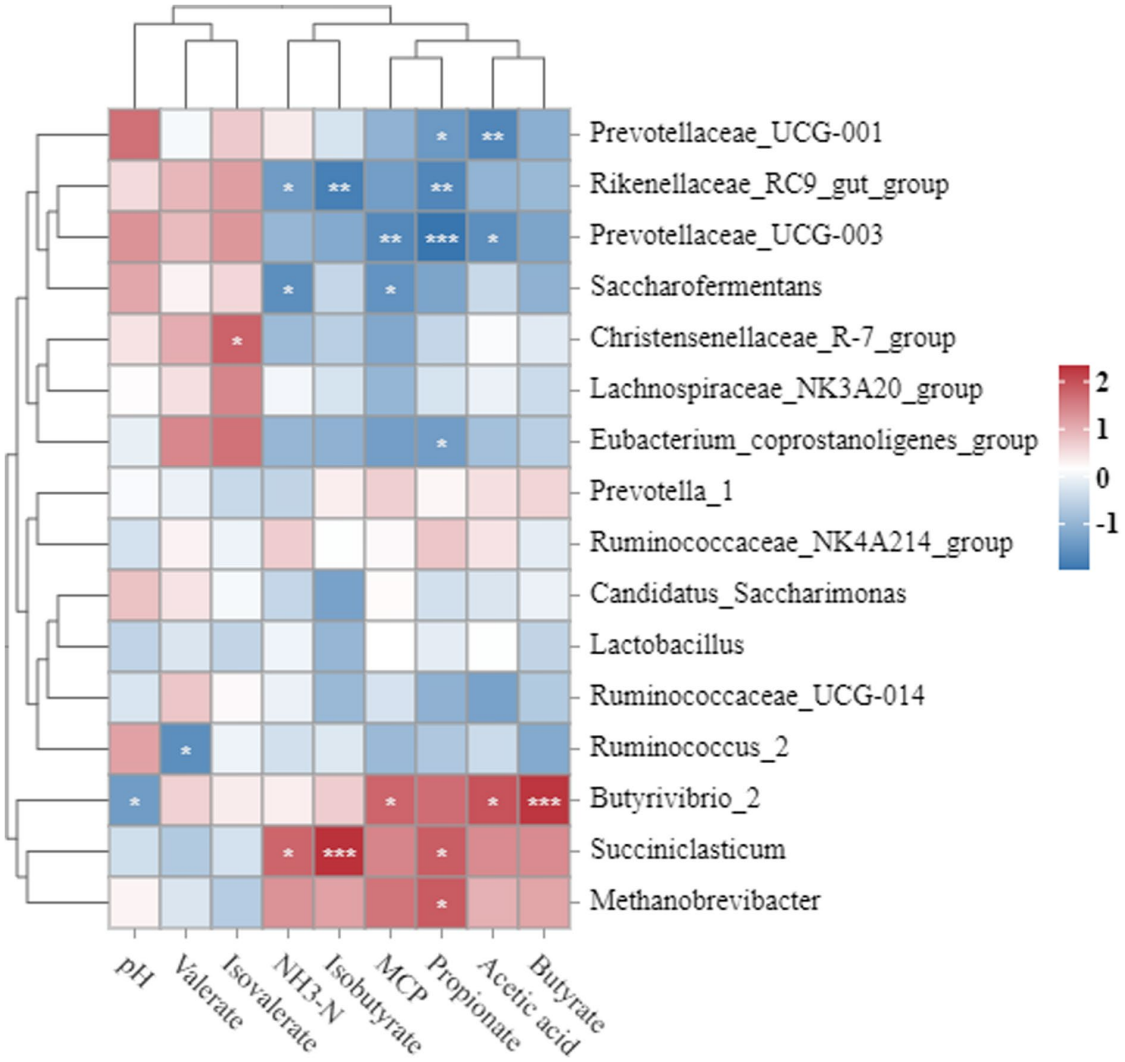
Correlation between rumen bacterial community and fermentation parameters in Simmental crossbred cattle fed bagasse and fermented sugarcane. Red and blue indicate strong correlations, 1 and 2 indicate positive correlations (dark red), −1 indicates negative correlations (dark blue), and white indicates weak correlations. Spearman correlation test, *p < 0.05, **p < 0.01, ***p < 0.001.

Correlations between rumen fermentation parameters and 16 rumen bacterial genera with a mean genus abundance greater than 1% were analyzed using Spearman’s correlation coefficient (r) (Figure 2). Ruminal pH was negatively correlated with *Butyrivibrio_2* abundance (*p* < 0.05). NH_3_-N was positively correlated with *Succiniclasticum* (*p* < 0.05) but negatively correlated with *Saccharofermentans* and *Rikenellaceae_RC9_gut_group* (*p* < 0.05). *Saccharofermentans* (*p* < 0.05) and *Prevotellaceae_UCG-003* (*p* < 0.01) were negatively correlated. Acetate was positively correlated with *Butyrivibrio_2* (*p* < 0.05) but negatively correlated with *Prevotellaceae_UCG-001* (*p* < 0.01) and *Prevotellaceae_UCG-003* (*p* < 0.05). Propionate content was positively correlated with *Succiniclasticum* and *Methanobrevibacter* (*p* < 0.05), but negatively correlated with *Eubacteriumcoprostanoligenes_group* (*p* < 0.05), *Prevotellaceae_UCG-001* (*p* < 0.05), *Prevotellaceae_UCG-00*3 (*p* < 0.001), and *Rikenellaceae_RC9_gut_group* (p < 0.01). Butyrate was positively correlated with *Butyrivibrio_2* (*p* < 0.001). Isobutyrate was positively correlated with *Succiniclasticum* (*p* < 0.001), but negatively correlated with *Rikenellaceae_RC9_gut_group* (*p* < 0.01). Valerate was negatively correlated with *Ruminococcus2* (*p* < 0.05). Isovalerate was positively correlated with *ChristensenellaceaeR-7grou*p (*p* < 0.05).

### 3.9. Prediction of rumen bacterial function

**Figure 3:**
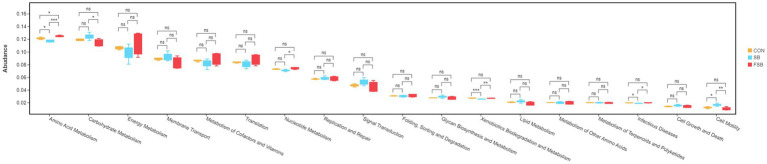
Functional prediction of rumen microbiota in Simmental crossbred cattle fed sugarcane bagasse and fermented sugarcane bagasse. *p < 0.05, **p < 0.01, ***p < 0.001.

The Tax4Fun gene function assessment method was used to predict the function of the rumen microbiota of Simmental crossbred cattle in the CON, SB, and FSB groups. Eighteen major pathways (relative abundance >1%) were enriched using the Tax4Fun prediction software in the 2-level KEGG pathway, of which six pathways were found to significantly differ among the three groups after two rounds of comparisons (*p* < 0.05; Figure 3). Interestingly, the relative abundance of the amino acid metabolites was significantly higher in the FSB group than in the SB (*p* < 0.001) and CON groups (*p* < 0.05); however, the relative abundance was significantly higher in the CON group than in the SB group (*p* < 0.05). The relative abundance of carbohydrate metabolism in the SB group was significantly higher than that in the FSB group (*p* < 0.05). The relative abundance of xenobiotic biodegradation and metabolism was significantly lower in the SB group than in the CON group (*p* < 0.001) and FSB group (*p* < 0.01).

## 4. Discussion

### 4.1 Growth performance and apparent digestibility of nutrients

Sugarcane bagasse is a by-product of sugarcane crushing, which is characterized by high fiber and lignin contents, and has a low degradation efficiency in the rumen. Direct feeding of ruminants with sugarcane bagasse results in the accumulation of large amounts of cellulose and lignin that cannot be rapidly digested in the rumen, encroaching on the limited physical space of the rumen and reducing the intake of dry matter in the feed to animals, eventually leading to a decrease in their growth performance (de Almeida et al., 2018). de Almeida et al. (2018) observed a linear decrease in dry matter intake, organic matter, and total digestible nutrient intake in Girolando dairy cows with an increase in the addition of bagasse dry matter. This study also found that feeding untreated sugarcane bagasse significantly reduced the dry matter intake, average daily gain and feed conversion ratio of Simmental hybrid cattle compared to the CON group. The degree of rumen filling is one of the most important factors influencing feed intake in cattle (Allen, 2000). Although the enzymatic treatment and microbial fermentation decreased the fibre content in the sugarcane bagasse, the lignin content in diet of the FSB group was 2.14% higher than that of the CON group (Table 2) and the apparent digestibility of NDF was slightly lower than that of the CON group (46.96 vs. 50.16%; Table 4). Compared to the CON group, the lower NDF digestibility and higher lignin content of the FSB group reduces digestion from the gastrointestinal tract, prolongs retention time in the rumen, increases rumen filling (de Almeida et al., 2018), resulting in a lower DMI in the FSB group.

Studies have shown that the use of enzymatic bacteria as fermenters can improve the quality, palatability, and digestibility of ruminant roughage (So et al., 2020). In this study, we found that the content of neutral detergent fiber, acid detergent fiber, and lignin in fermented sugarcane bagasse treated with a mixture of probiotics and cellulase decreased by 24.09, 28.25, and 20.67%, respectively, compared to untreated bagasse (Table 1). In addition, fermented sugarcane bagasse was found to significantly increase the dry matter intake and feed conversion efficiency of Simmental crossbred cattle, with a slight increase in average daily weight gain. So et al. (2021) showed that the administration of fermented sugarcane bagasse treated with *Lactobacillus casei* TH14, cellulase, and molasses significantly increased dry matter intake, daily weight gain, feed conversion efficiency, and apparent digestibility of dry matter, organic matter, neutral detergent fiber, and acid detergent fiber in native Thai cattle compared to untreated rice straw. High lignin content coulde reduces the palatability and efficiency of rumen microbial feed degradation. Cellulases disrupt the cross-linking of bagasse cell walls with lignin (So et al., 2022) and enhance bagasse degradation by ruminal bacteria. This finding is verified by the fact that the apparent digestibilities of dry matter, organic matter, and crude protein in the FSB group were significantly higher than those in the SB group, as well as a slight increase in the apparent digestibilities of NDF and ADF. The increase in the apparent digestibility of nutrients indicates that the retention time of surimi in the rumen is reduced (Kholif et al., 2022) and more nutrients can be accommodated in the rumen per unit time, which may be an important reason for the higher dry matter intake, daily weight gain, and feed conversion efficiency in the FSB group than in the SB group. The addition of mixed probiotics and cellulase improved the palatability and odor of fermented sugarcane bagasse (So et al., 2021), which may be another factor causing a significant increase in dry matter intake in the FSB group. Gunun et al. (2016) have reported that the apparent digestibility of NDF and ADF in sugarcane bagasse treated with urea or urea+calcium hydroxide was significantly higher than that in untreated sugarcane bagasse and rice straw. Andrade Andrade et al. (2016) have found that the apparent digestibility of DM, OM and NDF was higher in Lactobacillus-buchneri-treated sugarcane silage than in sugarcane silage without any treatment. These studies indicate that the change of the apparent digestibility of forages by different treatment methods. The change of apparent digestibility is related not only to the treatment methods but also to the nutritional characteristics of the forage itself. In this study, the FSB group showed lower growth performance and apparent digestibility of NDF than the CON group. Although cellulase and mixed probiotics can improve the nutritional value of fermented sugarcane bagasse, they can notfundamentally change the fact that sugarcane bagasse is a low-quality nutritious feed. This can patially explained the results in our study. No significant differences were found between the FSB and CON groups in terms of final body weight and feed conversion efficiency, suggesting that the replacement of hybrid giant napiers with 20% enzyme-treated bagasse (dry matter basis) did not affect the growth performances of Simmental crossbred cattle and breeding costs did not increase.

### 4.2. Ruminal fermentation

Ruminal pH is one of the most important indicators of rumen fermentation. In this study, the pH of the rumen fluid in the three treatment groups were similar, and the pH of 6.35–6.48 in the three treatment groups is within the optimal pH range of 6.0–7.0 for rumen fermentation conditions (Wanapat et al., 2011). The amount of saliva secreted as a buffer for ruminal pH is an important factor affecting ruminal pH. Goulart et al. (2020) revealed that the administration of bagasse to Nellore cattle significantly increased rumination and chewing time, while chewing activity was positively correlated with saliva production. In this study, the SB group tended to have higher pH values than the CON and FSB groups (*p* = 0.054), which may be related to the fact that the intake of sugarcane bagasse increases salivary secretion in Simmental crossbred cattle.

Sudweeks (1977) reported that cattle fed diets that increased chewing time and salivary flow had lower concentrations of SCFA due to a dilution effect and increased A/P. Although this study did not measure the chewing time and saliva flow of the experimental cows in each group, previous studies have confirmed that feeding sugarcane bagasse can increase the chewing time and saliva flow of ruminants. Additionally, in this study, the A/P ratio of the SB group is slightly higher than those of the CON and FSB groups. These results in this study tend to support findings of Sudweeks (1977). Therefore, the higher concentrations of Acetate, Propionate, and Butyrate found in the CON and FSB groups than in the SB group in this study may be due to the increased chewing time and salivary secretion by the sugarcane bagasse in the SB group. Compared to other conventional roughages, the ADL in sugarcane bagasse is blended and cross-linked with cellulose and hemicellulose (Wanapat and Cherdthong, 2009; Raffrenato et al., 2017), resulting in a decreased degradation of structural carbohydrates by rumen microorganisms (Castañón-Rodríguez et al., 2015). However, anaerobic fermentation may stimulate the hydrolysis of structural carbohydrates in sugarcane bagasse (Wang et al., 2018) and the addition of cellulase may directly disrupt the cell wall of sugarcane bagasse (So et al., 2020, 2021), which increases the degradation and utilisation of sugarcane bagasse by rumen microorganisms. In this study, the significantly lower apparent digestibility of DM, OM and CP in the SB group than in the CON and FSB groups supported the conclusions of the above researchers. In summary, the lower rumen degradation rate of sugarcane bagasse in the SB group can also explain the higher acetate, propionate, and butyrate concentrations in the CON and FSB groups than in the SB group. Valeric acid can be used as a marker to estimate the VFA uptake (Daniel et al., 2013). In the present study, there was no significant difference in the amount of valerate in the rumens of the three groups, suggesting that the ruminal volatile acid uptaked by the rumen epithelium is not affected by the different sugarcane bagasse diets. Therefore, with the same SCFA uptake by the rumen epithelium in the three treatment groups, the lower SCFA production in the SB group ultimately resulted in lower TVFA level in this group compared to the CON and FSB groups.

NH_3_-N is the most important source of nitrogen for microbial protein synthesis in the rumen. A concentration of 5 mg/dL of NH_3_-N is the minimum concentration required for microbial protein synthesis in the rumen (Satter and Roffler, 1975). Research has found that rumen NH_3_-N in the range of 15–30 mg/dL is most beneficial to improve microbial protein synthesis, nutrient digestion and free feeding of low quality roughage in ruminants fed (Wanapat et al., 2011; Gunun et al., 2016). In this study, although the NH_3_-N concentrations among the three treatment groups were not within the optimal range, the NH_3_-N concentrations of the CON and FSB groups were closer to the optimal concentration range than that of the SB group. Previous studies have shown that the NH_3_-N concentration in the rumen is positively correlated with the protein content and availability in the diet (Christensen et al., 1993; Gunun et al., 2016). The NH_3_-N content in the SB group could be related to the presence of fibrous material in bagasse, which blocks the degradation of proteins by rumen microorganisms. Ruminal microbial protein synthesis is influenced by the energy intake of ruminants when the amount of nitrogen and energy in the rumen become the main factors limiting microbial growth (Clark et al., 1992). de Almeida et al. (2018) revealed that microbial protein synthesis decreased linearly with the increase of the amount of bagasse added. The present study also revealed that the rumen MCP content in the SB group was significantly lower than that in the CON and FSB groups, which aligns with the changes of DM intake and apparent digestibility of nutrients in the three groups. This finding indicates that the MCP synthesis by rumen microorganisms in the SB group were hampered by the dificiency of nitrogen and energy, which ultimately led to a significantly lower MCP content in the SB group than in the other two groups.

### 4.3. Effect of rumen bacteria-related abundance

Bacterial diversity in rumen is influenced by dietary factors, such as the ratio of concentrates to forage (Tapio et al., 2017; Qian et al., 2018). Borchers and Pieler (2010) have found that the rumen OUTs and unique OUTs of yaks in the grazing group were higher than those in the grazing+supplementary feeding group. Lv et al. (2020) have reported that feeding diet with higher NDF content could increase the number of OUTs in the rumen of lambs. The similar changes in OUT and NDF levels in the three treatment groups in this study tend to be consistent with the view of Lv et al. (2020). The Chao and ACE indices of rumen bacteria in the FSB group were significantly lower than those in the other two groups, and the ACE index was significantly lower than that in the SB group. Such finding indicates that the mixed enzyme bacterial treatment reduced the diversity and abundance of rumen bacteria, which may be related to the lower NDF content in the FSB group (Cui et al., 2019).

At the phylum level, the highest abundance in the three treatment groups was the *Firmicutes*, followed by the *Bacteroidetes*, which is similar to the results of Pang et al. (2022) but opposite to those of Mu et al. (2019). Species in the *Firmicutes* are rich in cellulases and hemicellulases (Gharechahi and Salekdeh, 2018) which play important roles in the degradation of fibrous materials (Evans et al., 2011). The higher levels of *Firmicutes* in the three treatment groups suggest that the number of bacteria involved in rumen fiber degradation is higher than the number of bacteria involved in starch degradation (Lv et al., 2020). However, the *Bacteroidetes* has been found to play a greater role in structural carbohydrate degradation (El Kaoutari et al., 2013), such as the degradation of lignin, than the *Firmicutes*, and a lower ratio of the *Firmicutes* to *Bacteroidetes* facilitates lignin degradation in the rumen (Mu et al., 2019). Based on the lower *Firmicutes* to *Bacteroidetes* ratio in the SB group in this study, the addition of bagasse with higher lignin content stimulated the growth of lignin degradation-related flora in the rumen. Ran et al. (2021) demonstrated that replacing maize silage with sorghum silage with higher NDF and ADL contents significantly increased the abundance of *Kiritimatiellaeota* in the rumen. This result aligns with that of the present study, which showed that sugarcane bagasse with higher NDF and ADL contents significantly increased the abundance of *Kiritimatiellaeota* in the rumen of Simmental crossbred cattle. According to Zhang et al. (2021), the lower DMI in the SB group may be responsible for the higher abundance of *Kiritimatiellaeota* in the ruminal fluid of this group. *Planctomycetes* are important players in the global carbon and nitrogen cycles and can function as the sole carbon source with high-molecular-weight polysaccharides that degrade complex carbon substrates (Wiegand et al., 2018). Therefore, the higher *Planctomycetes* levels in the SB group may be related to the fact that the addition of sugarcane bagasse with high NDF and ADL contents stimulated the growth of bacteria associated with this phylum.

At the genus level, the dominant bacteria in the rumen were *ChristensenellaceaeR-7group*, *Prevotella1*, *RikenellaceaeRC9gutgroup*, and *Succiniclasticum*. *Christensenellaceae_R- 7_group* belongs to the *Firmicutes* (Waters and Ley, 2019) which are mainly involved in the degradation of cellulose and hemicellulose in the rumen (Evans et al., 2011; Ran et al., 2021) and usually have a high abundance in the rumen of ruminants fed diets with a high fiber content (Thoetkiattikul et al., 2013). The higher abundance of *Christensenellaceae_R-7_group* in the SB group may be related to the presence of 20% bagasse containing less degradable fiber material in this group (Pang et al., 2022). *Succiniclasticum* is an important member of the core rumen microbiota, is involved in starch degradation, and is a major contributor to the conversion of succinic acid to propionic acid in the rumen (Ran et al., 2021), with a higher abundance in the rumen of ruminants fed high-energy and high-concentrate diets (Bi et al., 2018; Liu et al., 2019). The lower abundance of *Succiniclasticum* in the SB group is consistent with the increase in NDF and ADL content reported by Ran et al. (2021). The lower *Succiniclasticum* abundance levels in the SB group were similar to the level reported by Ran et al. (2021), who found that increased sorghum silage with higher NDF and ADL content reduced *Succiniclasticum* abundance levels in the rumen of lactating cows. The present study also revealed that the *Succiniclasticum* abundance was significantly higher in the FSB group than in the CON and SB groups. This finding may be related to the use of cellulase to destroy the cell wall of sugarcane bagasse, resulting in nonstructural carbohydrates in sugarcane bagasse being more easily degraded by ruminal microorganisms. However, the specific reason for this observation requires further investigation. *Previotellaceae_UCG-003* is involved in the degradation of plant cell wall polysaccharides. Borchers and Pieler (2010) found that grazing increased the abundance of *PreviotellaceaeUCG-003* in the rumen of cattle compared to that in supplemented cattle. This finding suggests that the abundance of *Previotellaceae_UCG-003* in the rumen was positively correlated with the fiber level of the diet, as indicated by the higher abundance of *Previotellaceae_UCG-003* in the SB group. *Rikenellaceae_RC9_gut_group* belongs to the family, *Rikenellaceae*, and plays an important role in fiber digestion. A positive correlation exists between the abundance of *Rikenellaceae_RC9_gut_group* and dietary fiber content (Wang et al., 2019). A previous study showed that the relative abundance of the *Rikenellaceae_RC9_gut_group* in the rumen decreased by 69.8% when the neutral detergent fiber content of the diet was reduced from 39.7 to 30.9% (Zened et al., 2013). Therefore, the higher NDF content in the SB group can be used to explain the higher abundance of *Rikenellaceae_RC9_gut_group* in the rumen fluid of this group. *Saccharofermentans*, a member of Firmicutes, has a strong cellulolytic activity and plays an important role in the degradation of rumen lignin. The higher abundance of *Saccharofermentans* in the SB group may be related to the inclusion of bagasse with higher lignin content in the diet of this group. *Eubacterium_coprostanoligenes_group* belongs to the family, *Rumenococcaceae*. *Eubacterium coprostanoligenes* belongs to the family, Eubacteriidae, which degrades starch, xylan, and other complex polysaccharides (Chassard et al., 2012). Similar to *Saccharofermentans*, the higher abundance of *E. coprostanoligenes* in the SB group may be related to the higher structural carbohydrate content in the diets of the SB group. However, high-concentrate or high-energy diets may increase the abundance of *E. coprostanoligenes* (Wang et al., 2020; Zhang et al., 2022). The differences between the above studies may be related to differences in animal species and feed components.

### 4.4. Indicator species analysis

Indicator species analysis helps identify potential indicator species in different groups. The dominant genera in the CON, SB, and FSB groups were *Lactobacillus*, Rikenellaceae_RC9_gut_group, and *Succiniclasticum*, respectively. *Succiniclasticum* is involved in starch degradation and plays a major role in the conversion of succinic acid to propionic acid in the rumen (Ran et al., 2021). *Rikenellaceae_RC9_gut_group* plays an important role in rumen fiber digestion, and an increase in dietary fiber content can promote its growth (Zened et al., 2013; Wang et al., 2019). Therefore, we believe that *Rikenellaceae_RC9_gut_group* is an indicator species for the bagasse group and *Succiniclasticum* is the indicator species for the FSB group. No significant difference was found among the three *Lactobacillus* treatments (*p* = 0.052), and owing to the large variation within groups, whether *Lactobacillus* can be used as an indicator species in the CON group remains to be further investigated.

### 4.5. Association between rumen microorganisms and rumen fermentation parameters

Ration-induced changes in rumen microbial community structure are associated with changes in ruminal fermentation parameters. The present study revealed the effect of partial substitution of sugarcane bagasse and fermented sugarcane bagasse in Simmental crossbred cattle on the rumen microbiota and rumen metabolic phenotypes. Correlation analysis revealed that propionate content was positively correlated with the relative abundance of *Succiniclasticum* but negatively correlated with the relative abundance of *Prevotellaceae_UCG-003* and *Rikenellaceae_RC9_gut_group*. The NH_3_-N content was positively correlated with the relative abundance of *Succiniclasticum* but negatively correlated with the relative abundance of *Rikenellaceae_RC9_gut_group*. MCP content was negatively correlated with *Prevotellaceae_UCG-003*. Propionate is the main substrate for conversion to glucose during gluconeogenesis and is the main source of energy for animals (Murad and Azzaz, 2019). Microbial proteins are an important source of high-quality protein for ruminants, and the nitrogen (NH_3_-N) content and energy utilization efficiency in the rumen are closely related to microbial protein synthesis (Clark et al., 1992; de Almeida et al., 2018). Therefore, the higher abundance of *Succiniclasticum* and lower abundance of *Prevotellaceae_UCG-003* and *Rikenellaceae_RC9_gut_group* in the rumen fluid of the CON and FSB groups may be associated with better daily weight gain in Simmental crossbred cattle in the CON and FSB groups.

### 4.6. Prediction of microbiota function

In this study, we used Tax4Fun to predict the effects of bagasse and fermented sugarcane bagasse on the rumen microbial communities of Simmental crossbred cattle. The data showed that genes involved in amino acid metabolism in the 2-level KEGG pathway were significantly enhanced in the rumen of Simmental crossbred cattle in the FSB group compared to those in the SB group. This result indicates that the rumen microbiota of Simmental crossbred cattle fed fermented sugarcane bagasse produced large amounts of proteins to provide the host with raw materials, such as proteins, which in turn sustained life and normal metabolism. The genes involved in carbohydrate metabolism in the 2-level KEGG pathway were significantly enhanced in the rumen of Simmental crossbred cattle in the SB group compared to those in the FSB group; however, the genes involved in the degradation and metabolism of foreign substances were significantly weaker. This finding may imply that the rumen microbes of Simmental crossbred cattle fed sugarcane bagasse were more capable of degrading complex fibrous material but less capable of integrated degradation and utilization of feed nutrients than the CON and FSB groups, and their energy delivery to the host through rumen microbial action was lower. The results of this study may not be representative of the actual function of rumen bacteria, and the mechanism of action of these genes in simpler crossbred cattle fed with bagasse and fermented sugarcane bagasse must be further elucidated using macrogenomic analysis.

## 5. Conclusion

Substitution of 20% sugarcane bagasse for hybrid giant Napier reduced dry matter intake, daily weight gain, and apparent nutrient digestibility and increased the relative abundance of bacterial genera associated with fiber metabolism in the rumen of Simmental crossbred cattle. Replacing grass with 20% fermented sugarcane bagasse had no adverse effects on growth performance, apparent nutrient digestibility, or rumen core flora abundance in Simmental crossbred cattle. Therefore, the combined fermentation of sugarcane bagasse with enzymatic bacteria is feasible as roughage for Simmental crossbred cattle.

## Data availability statement

The data presented in the manuscript has been made available in the NCBI database with accession PRJNA1033327.

## Ethics statement

The animal study was approved by Welfare and Ethics Committee of the College of Animal Science at Xichang University (Approval No. xcc2022031). The study was conducted in accordance with the local legislation and institutional requirements.

## Author contributions

YJ: data analysis and writing of the first draft. YH, HL, LW, BC, and YZ: feeding management and sample collection. KD and NZ: providing the test site and test animals. AL: experimental protocol design, data analysis, provided project funding, proofreading, and finalization of the paper. All authors used contributed to this article and agreed to submit this version.

## Footnotes

^1^http://drive.com/uparse/
